# Cross-time analysis of physical exercise and traditional gender concepts: Chinese women in the CGSS 2013 and 2023

**DOI:** 10.1186/s12889-026-27574-z

**Published:** 2026-04-28

**Authors:** Lei Sun, Ping Fang

**Affiliations:** 1 Nanjing University of Chinese Medicine, No.138 xianlin Road, Nanjing, 210023 China; 2 School of Sports Science, Nanjing Normal University, Wenyuan Road 1, Nanjing, 210023 China

**Keywords:** China, Physical exercise, Traditional gender concepts, CGSS, Empirical analysis

## Abstract

**Objectives:**

Within the context of deeply ingrained traditional gender concepts influenced by Chinese cultural background, women in China persistently confront widespread issues of gender inequality. Although socioeconomic and demographic characteristics, social hierarchy, and physical exercise are acknowledged as significant factors, this study specifically examines the relationship between physical exercise and women’s traditional gender concepts.

**Methods:**

Utilizing data from the 2013 and 2023 waves of the Chinese General Social Survey, this study applied multiple ordinary least squares (OLS) regression analyses to assess the impact of physical exercise on women’s traditional gender concepts. To ensure the robustness of the findings, propensity score matching techniques were employed. Furthermore, instrumental variable two stage least squares (IV-2SLS) was implemented to mitigate potential endogeneity concerns stemming from reverse causality or omitted variable bias.

**Results:**

(1) In 2013, the percentage of women participation in physical exercise was lower than that of women who did not participate. Conversely, by 2023, this trend had reversed. The sample primarily consisted middle-aged and older women, individuals with agricultural household registration, married women, those possessing educational backgrounds at the primary, junior, or general high school levels, as well as women belonging to the middle social hierarchy. (2) From the standpoint of traditional gender concepts, in 2023, among young and middle-aged women—regardless of agricultural or non-agricultural household registration status—who were married, residing in the eastern and western regions, and possessing educational qualifications ranging from junior school and general high school to associate and bachelor degrees obtained through regular higher education, as well as those situated within the 3rd to 7th tiers of the social hierarchy, the degree of traditional gender concepts was markedly lower compared to 2013. Conversely, older women exhibited a relative increase in adherence to these traditional gender concepts; Regarding participation in physical exercise, in 2023, across young, middle-aged, and older female cohorts with both agricultural and non-agricultural household registrations, who were married, living in the middle, eastern, and western regions, and holding educational levels from junior school and general high school to associate and bachelor degrees attained via regular higher education, and positioned within the 1st to 6th social hierarchy levels, the frequency of participation in physical exercise was higher than that recorded in 2013; (3) OLS estimates indicated that participation in physical exercise significantly reduced traditional gender concepts among women in both 2013 and 2023, with the magnitude of this effect being greater in 2023; (4) IV-2SLS estimations confirmed robustness, revealing a significant negative effect in 2023 after accounting for endogeneity; Advanced age, possession of an agricultural household registration, and lower levels of educational attainment were associated with more pronounced traditional gender concepts, whereas higher educational attainment served to attenuate these tendencies.

**Conclusions:**

This study demonstrates that participation in physical exercise attenuates traditional gender concepts among women in China, with a more pronounced impact observed in 2023. Physical exercise is significantly associated with the diminution of traditional gender concepts established for the year 2023, whereas the effect in 2013 was found to be statistically insignificant. Additionally, factors such as life stage, household registration status, and schooling were shown to affect traditional gender concepts . These findings underscored the significance of physical exercise in advancing gender role modernization and provide empirical support for policies aimed at increasing women's participation in physical exercise.

## Introduction

The analysis and evaluation of traditional gender constructs serve as essential metrics for gauging a nation’s progress in addressing gender disparities, as well as for identifying the extent to which its population maintains conventional, non-modernized perspectives on gender. This indicator has exerted a pivotal influence on the political, social, and economic emancipation of women worldwide, with far-reaching implications for the lives of innumerable individuals. In the conventional social division of labor based on gender, patriarchal traditions have become deeply ingrained within societal consciousness, whereby men predominantly assume roles within the public sphere, whereas women are primarily associated with responsibilities within the domestic sphere. The perspective of the life course indicates that the formation and differences of gender concepts are often related to the key moments and events that individuals encounter during their life journey, such as marriage [1], urbanization [2], and participation in the labor market [3]. Traditional gender concepts are still prevalent [4–7]. Wives with relatively high economic independence and decision power hold the least traditional gender concepts, while those with high economic dependence and decision power exhibit the most traditional gender concepts [8]. This shift signifies a transition from the conventional paradigm of “men working outside the home and women managing domestic affairs” to a contemporary egalitarian discourse [9]. The continuous challenge to traditional gender concepts that women experience greater benefits from the concept of gender equality, which constitutes a significant milestone in the progression of human civilization. Women are gradually entering the public domain that was once dominated by men, thereby gradually challenging and reshaping the traditional definition of gender roles, which pave the way for the popularization of the concept of gender equality. Nevertheless, substantial challenges persist in the actual dissemination and implementation of open equality gender concept. Notably, advancements in gender equality have been unevenly distributed, with marked disparities existing among diverse female populations in China, especially across urban–rural divides, educational attainment, and socioeconomic status [10]. The findings provide novel insights into gender disparities in the intergenerational transmission of education. Specifically, when wives possess higher educational attainment than their husbands, respondents tend to hold more open egalitarian gender concepts.

As a comprehensive national initiative, the “Healthy China” strategy is aimed at improving the overall health and well-being of the Chinese population, with the core goal of safeguarding people’s health as the fundamental purpose and integrating health concepts into the whole process of public policy formulation and implementation. Furthermore, the growing prevalence of the national fitness initiatives has substantially contributed to the enhanced involvement of women in physical exercise throughout China. It is noteworthy that the “Healthy China 2030” blueprint identifies women as a key target group for promoting physical exercise [11]. Women’s mass sport is a production of the interaction of socioeconomic and cultural contexts, being widely and deeply determined by the new social forces and the social situation of women [12]. Physical exercise—a routine practice integrating physical exercise, social interaction, and cultural meaning—provides a useful theoretical perspective for exploring the micro-level development of gender beliefs. The former study indicated that stereotype-consistent sports activities led to more masculine evaluations of targets, while stereotype-inconsistent ones reversed gender evaluations, particularly for women [13]. It should be pointed out that Tai Chi may be further leveraged to explore and realize its potential in reshaping gender structures and reducing gender antagonism [14]. Baed on social cognitive theory, men tended to rate sports as significantly more masculine than women [15]. However, the reality is that the frequency of women’s engagement in physical exercise have shown an increasing trend over the past decade [16], which can be regarded as a manifestation of contemporary lifestyles. Physical exercise may reduce traditional gender concepts through multiple interrelated pathways. Grounded in social cognitive theory, participation in regular physical exercise enhances individuals’ perceived competence and self-efficacy, which empowers them to challenge restrictive gender norms. Meanwhile, embodiment theory suggests that active bodily practice fosters bodily empowerment, enabling individuals to transcend passive, gendered bodily expectations. The developmental theory of embodiment shapes the experience of embodiment, a construct that is strongly correlated with body esteem and body appreciation [17]. Importantly, sport has historically served as a critical site for the construction and performance of gender identities [18], hold positive implications for women empowerment and the reconfiguration of gender norms [19]. As of now, little research has focused on the implications of physical exercise on traditional gender concepts. There is a lack of empirical investigations based on large-scale, representative samples focusing on the general women population in China.

Accordingly, this study uses data from the 2013 and 2023 waves of the Chinese General Social Survey (CGSS) to systematically examine the influence of physical exercise on traditional gender concepts among Chinese women. The present study therefore adopts rigorous econometric models to more accurately identify the relationship between physical exercise and traditional gender concepts. Against the backdrop of rapid economic and social transition in contemporary China, the profound shifts in gender roles provide a unique context to explore critical issues related to physical exercsie participation and gender equality.

## Literature review

### The traditional gender concepts and physical exercise

The theoretical underpinnings of both sociology and gender studies posit the notion that gender is not an innate, biologically determined construct; rather, it is a social and cultural construct [20]. China traditional gender concepts during the past century began at an extreme, with the Confucianism of the Imperial era [21]. During this period, women were systematically subordinated through a confluence of cultural, familial, and legal institutions. The Confucian doctrine of obedience to one’s father before marriage, to one’s husband during marriage, and to one’s sons after the husband’s death strictly regulated women’s conduct and legitimized male dominance over women [22]. These included the Confucian Five Relationships (wu lun), the doctrine of filial piety, traditional yin-yang ideology that prescribed passive female roles, clan norms governing marital relations, and formal state laws. Collectively, these structures confined women to a subordinate social status, with severely restricted access to education, employment, and political participation. In the Chinese context, traditional gender concepts have commonly been expressed through expectations that men prioritize paid work, women prioritize family roles, men possess greater competence, and women hold secondary status in marriage and employment [5]. These capture central normative expressions of traditional gender concepts in contemporary China, particularly attitudes toward gendered division of labor, male superiority, women’s marriage-oriented roles, and women’s secondary labor-market status. These dimensions are widely recognized as fundamental to traditional gender norms in China, where some other dimensions remain salient. It is important to note that this measure does not encompass all possible dimensions of traditional gender concepts. Traditional gender concepts are multidimensional, and the present measure does not cover several relevant domains. It does not directly assess attitudes toward household decision-making power, including control over finances and major family decisions, which are important indicators of intra-family gender hierarchy [23]. It also excludes attitudes toward domestic violence, reproductive autonomy, son preference, and women’s participation in leadership or public life. As such, our operationalization should be interpreted as focusing on a specific but central dimension of traditional gender concepts—namely, beliefs about gender roles and relative abilities in family and labor market contexts. China traditional gender concepts are not merely a social injustice but a pervasive barrier to sustainable development, with detrimental consequences that resonate across economic, social, and individual [24, 25]. Practically, from women economic wellbeing and financial autonomy, through labour force participation and continuity of employment, to occupational attainments and economic rewards, the analysis confirms the existence of distinctive profiles of gender inequality [26]. These structural forms of gender inequality, in turn, shape individuals’ perceptions and attitudes toward gender roles. Individuals gradually accept and internalize a spectrum of gender concepts, ranging from traditional to egalitarian, through socialization contexts including the family, school, and mass media [27]. Previous studies have indicated that egalitarian gender concepts, which advocate for the equitable distribution of responsibility between men and women in both household affairs and paid employment, have the potential to enhance the quality of life and promote greater happiness for both genders [28, 29]. Importantly, the traditional gender concepts are being torn apart, while more open concepts of gender equality are constantly emerging.

Embodiment Theory posits that human cognition, attitudes, and social perceptions are not abstract, but are shaped and constructed through physical experiences and bodily interactions with the environment. Specifically, the body is not merely a “carrier” of social norms, but an active agent that reconstructs individual perceptions—including gender norms—through physical practice. This theory provides a key theoretical lens for understanding the relationship between physical exercise and gender concepts: physical exercise, as a purposeful, repetitive physical activity, can reshape individuals’ bodily experiences, thereby challenging the internalized gender concepts that are embedded in bodily practices (e.g., the perception that “physical strength is a masculine trait”). Existing studies have explored the correlation between physical exercise and gender attitude changes [18, 19], but few have explicitly articulated the underlying mechanism. From the perspective of embodiment theory, these correlations can be explained by the bodily experiences brought by physical exercise: for example, female participation in strength training can break the bodily stereotype of “femininity as weak”, and long-term physical exercise can help individuals decouple their self-perception from traditional gender concepts embedded in the body.

Women participation in physical exercise was particularly relevant in the Chinese context, as women’s sport also presented a range of contradictions [30]. Practices such as foot-binding severely restricted women’s physical mobility, and, much as in Western cultures, women were socially excluded from physical exercise [31]. While general gender studies have clarified the formation and reinforcement mechanisms of traditional gender concepts, the specific behavioral pathways through which individuals can deconstruct these internalized norms remain under-explored. To fill this gap, this study adopts the perspective of embodiment theory, which provides a theoretical bridge between general gender norms and the specific role of physical exercise—specifically, it explains how physical exercise, as a purposeful physical or behavioral practice, can reshape individuals’ gender-related perceptions and challenge traditional gender concepts. With the founding of the People’s Republic of China came the emergence of a new sports culture and the promotion of a ‘’mass sports participation’’ policy. Women and girls were regarded as a vital force in national reconstruction and defense, ‘athletic women became visible human beings and entered onto the stage of New China’ [22]. National funding was directed at elite sport to bolster Chinese women’s success at international sports. In the realm of competitive sports, female athletes have constituted ‘’women hold up half the sky,‘’ embodying fortitude and resilience, while their femininity is often overlooked [32]. In the early years of the People’s Republic of China, investment in women’s leisure and sporting activities remained inadequate. Nowadays, China has invested a large amount of funds in the field of national fitness and health among women. Furthermore, physical exercise can serve to develop a critical awareness of gender regulation [33, 34]. Engaging in physical exercise pushes women to transcend physical and mental limits. Women who participate in sports are often perceived as less feminine, and existing research has documented the pressures faced by young female athletes to conform to a more masculine physique [35], thereby ‘strong, tough and beautiful women athletes’ comes the ‘emergence of a potentially new form of femininity that refuses to cede physical strength and sporting excellence to men. Their success in transcending traditional gender concepts further enables them to challenge prevailing stereotypes about women’s physical and psychological capacities. It creates an upsurge in interest for women to participate in physical activity as part of their daily lives. It is evident that physical exercise can challenge traditional gender concepts. Although a growing body of literature has investigated the determinants of individuals’ gender concepts, limited attention has been paid to the role of physical exercise in shaping women’s traditional gender concepts. Hypothesis 1: Overall, physical exercise will reduce the inherent stereotypes of traditional gender concepts.

### The factors influencing traditional gender concepts through socio-demographic variables

As societal norms advance toward greater gender equality, the strong ideological interaction between spouses provides an important channel for the diffusion of egalitarian gender beliefs [36]. To date, the majority of gender equality initiatives have tended to adopt a one-sided approach to gender equality: focusing on the need to eliminate barriers faced by girls and women, while traditional gender concepts should adapt to changes driven by the revival of women’s power [37]. Economic development was associated with liberal egalitarian and egalitarian essentialist attitudes, both of which fostered ideological reform toward gender equality concepts. Ideologies combining gender essentialist and egalitarian perspectives have increasingly supplanted traditional gender-inegalitarian views, even in societies that maintain institutional support for gendered separate spheres. Nevertheless, a significant minority of couples challenge dominant gender norms, particularly in their paid work arrangements. Bornatici et al. (2025) advocated policy reforms to establish supportive institutions that empower couples to align their behaviors with their gender concepts, thereby paving the way for greater gender equality in the future [38]. Gender concepts are widely viewed as being in a constant state of flux, continuously shaped and reshaped through their dynamic interplay with social structures—including but not limited to educational systems, labor markets, and family institutions [39–41]. While significant progress has been achieved in advancing gender equality, diversity, and inclusion in the workplace, education, and society. In line with this, the present study demonstrates that higher educational attainment serves as a robust predictor of stronger gender-egalitarian attitudes among women, since education directly empowers them to challenge traditional gender-role inequalities [42]. Respondents with lower educational attainment and male respondents were more likely to hold non‑egalitarian gender ideologies. Thus, educational disparities play a crucial role in challenging traditional gender concepts. Longitudinal studies have consistently shown that women who maintain continuous employment are most likely to hold egalitarian gender attitudes. Furthermore, women who re-enter the workforce following childbirth may develop even more egalitarian concepts, potentially as a result of navigating the competing demands of paid work and family responsibilities [43]. Yet the “consensus” documented by Aafke Komter more than three decades ago—one in which both men and women endorsed male primacy and viewed it as natural for women to embrace housework and for men to pursue professional aspirations—has weakened among college-educated individuals in the upper-middle class [44]. The findings demonstrated that age, educational attainment, country of education, and wives’ employment status did not emerge as significant predictors of egalitarian gender concepts after controlling for acculturation level, while all predictor variables were considered together, they explained 33% of the variance in gender role concepts–a proportion higher than that explained by any single predictor variable alone [45]. In addition, social hierarchy was measured as a continuous 1–10 scale capturing respondents’ subjective perception of their social position in the broader social structure, independent of objective material resources, social hierarchy reflects subjective social positioning. Therefore, Hypothesis 2: Demographic variables (life stage, Hukou, marital status, locality, schooling and social hierarchy) are associated with physical exercise and traditional gender concepts.

## Methods

### Measure of variables

#### Physical exercise

Physical exercise was categorized dichotomously (no participation = 0, participation = 1) according to responses to the item: “Over the past year, have you frequently participated in physical exercise during your leisure time?” Participants who responded “never” were assigned a score of 0, whereas all other responses were assigned a score of 1.

### Traditional gender concepts

The dependent variable in this article were the traditional gender concepts. The higher the score, the more unequal and unmodern the gender concepts were. Conversely, the lower the score, the more modern and equalization it tended to be. In this study, traditional gender concepts are conceptualized as normative beliefs regarding appropriate roles, abilities, and social positions of men and women, particularly in the domains of family and the labor market. To operationalize this construct, CGSS has been widely used in prior research to measure gender role concepts in China. We operationalized gender concepts using four items from the CGSS: (a) “Men should prioritize career over family; women, family over career.“; (b) “Men are naturally more capable than women.“; (c) “Marrying well is preferable to career success for women.“; and (d) “Women should be laid off first during economic downturns.” Responses were captured on a five-point ordinal scale (disagree = 1, relatively disagree = 2, neutral = 3, relatively agree = 4, completely agree = 5). To accommodate dimensional diversity across items, we applied factor analysis to derive composite scores. Table 1 presented the psychometric properties: Cronbach’s alpha values of 0.681 (2013) and 0.727 (2023) confirmed acceptable internal consistency, while the Kaiser-Meyer-Olkin (KMO) statistics of 0.703 (2013) and 0.744 (2023) validated the appropriateness of factor analytic procedures. By conducting Bartlett’s test of sphericity and KMO test, it was determined whether factor analysis was appropriate. This standardization method was achieved by subtracting the minimum factor value from each original factor value, then dividing the result by the range of the factor values and finally multiplying by 100. Its advantage lied in maintaining the original distribution characteristics of the data while converting all factor values to the same scale, making the gender concept factor values easier to compare, interpret and analyze.

**Table 1:** Factor analysis results of traditional gender concepts

Analysis of traditional gender concepts factor loading in 2013			
Item	Mean	SD	Traditional gender concepts factor loading
Men should prioritize career over family; women, family over career	3.417	1.139	0.7387
Men are naturally more capable than women	2.960	1.181	0.8014
Marrying well is preferable to career success for women	3.176	1.137	0.6893
Women should be laid off first during economic downturns	2.153	0.986	0.6294
Root of characteristic	2.049		
KMO	0.703		
Bartlett’s test for sphericity	*P* < 0.001		

Analysis of traditional gender concepts factor loading in 2023			
Item	Mean	SD	Traditional gender concepts factor loading
Men should prioritize career over family; women, family over career	3.105	1.426	0.7855
Men are naturally more capable than women	2.785	1.380	0.8027
Marrying well is preferable to career success for women	3.082	1.372	0.7278
Women should be laid off first during economic downturns	1.877	1.107	0.6394
Root of characteristic	2.201		
KMO	0.744		
Bartlett’s test for sphericity	*P* < 0.001		

#### Concomitant variables

Based on established literature and data availability, we controlled for seven socio-demographic and socio-economic indicators from the CGSS (Table 2). Life stage was categorized as young (15–44 years = 1), middle-aged (45–59 years = 2), or older (≥ 60 years = 3). Hukou was coded dichotomously (agricultural = 0, non-agricultural = 1) based on current household registration. Marital status was binary (unmarried = 0, married = 1). Geographic locality distinguished eastern, middle, and western regions. Schooling was operationalized across 13 hierarchical levels. Social hierarchy was measured on a 10-point self-ranking scale, as subjective social hierarchy had been theoretically and empirically linked to gender value orientations beyond objective socioeconomic indicators. This measure reflected individuals’ perceived social position rather than purely objective income, education, or occupation. We preferred it over objective composite indices because our theoretical framework emphasizes perceived status constraints on gender role negotiation rather than material resource availability. We also applied the multicollinearity test. All VIF values were well below 10, confirming no multicollinearity between these two variables (Table 3).

**Table 2:** Variables definition

Type	Variable	Variable declaration
Dependent variable	Traditional Gender concepts	Score for traditional gender concepts
Independent variable	Physical exercise	Not participation = 0; participation = 1
Control variables	Life stage	Young (15–44 years old) = 1; Middle-aged (45–59 years old) = 2; Older (60 years old and above) = 3
	Hukou	Agricultural household registration = 0; Non-agricultural household = 1
	Marital status	Unmarried = 0; Married = 1
	Locality	East = 1; Middle = 2; West = 3
	Schooling	No formal education = 1; Private school and literacy class = 2; Primary school = 3; Junior school = 4; Vocational high school = 5; General high school = 6; Technical secondary school = 7; Technical school = 8; Associate degree (adult higher education) = 9; Associate degree (regular higher education) = 10; Bachelor (adult higher education) = 11; Bachelor degree (regular higher education) = 12; Graduate student or above = 13
	Social hierarchy	1 point = 1; 2 points = 2; 3 points = 3; 4 points = 4; 5 points = 5; 6 points = 6; 7 points = 7; 8 points = 8; 9 points = 9; 10 points = 10

**Table 3:** Test for multicollinearity

Variable	VIF (2013)	VIF (2023)
Physical exercise	1.24	1.15
Life stage	1.15	1.19
Hukou	1.46	1.31
Marital status	1.01	1.00
Locality	1.11	1.12
Schooling	1.56	1.42
Social hierarchy	1.07	1.04

#### Data sources and processing

Data were drawn from CGSS database, which was administered in 16 provinces, autonomous regions, and centrally governed municipalities across 199 communities. The survey instrument includes more than 400 variables measuring education, economic status, behavior, and attitudes. We restricted our analysis to women respondents; after removing observations with incomplete data, the final analytic sample consisted of CGSS 2013 (4331) and CGSS 2023 (2143).

#### Analytic strategy

We specify a multiple linear regression model to assess the relationship between traditional gender concepts and physical exercise:

Traditional gender concepts$\:i$ = *β0* + *β1* Physical exercise$\:i\:$+$\:\:\sum\:_{i=2}^{n}\beta\:i$X$\:i\:$ + ε$\:i$

where *X* denotes the set of control variables. To verify the robustness of our results, we implemented Propensity Score Matching (PSM). Additionally, to address potential endogeneity concerns, this study employs an instrumental variable approach using two-stage least squares (IV-2SLS).

## Results

### Descriptive statistics

As demonstrated in Table 4, the descriptive statistical analysis indicated that the proportion of women physical exercise was 42.420% (2013) and 54.410% (2023). Moreover, it was observed that the proportion of married, middle-aged (45–59 years old) and older (60 years old and above), and agricultural household registration was relatively high in both 2013 and 2023.With regard to locality, the number of subjects in the middle, western and eastern regions was found to be comparable. The majority of the samples were concentrated in the educational background of primary school, junior school and general high school, accounting for 70.560% (2013) and 71.100% (2023) of the total sample. With regard to the social hierarchy, the majority of participants concentrated on the 1–7 point range, with a significant proportion focusing on the 5-point category, a small number of individuals attained high scores.

**Table 4:** Descriptive statistics of variables

Variable	Classification	2013			2023		
		Freq	%	Cum	Freq	%	Cum
Physical exercise	Not participation	2494	57.580	57.580	977	45.590	45.590
	Participation	1766	42.420	100.000	1166	54.410	100.000
Life stage	Young (15–44 years old)	695	16.050	16.050	520	27.270	27.270
	Middle-aged (45–59 years old)	1571	36.270	52.320	746	38.810	59.080
	Older (60 years old and above)	2065	47.680	100.000	877	40.920	100.000
Hukou	Agricultural household registration	2734	63.160	63.130	1536	71.680	71.680
	Non-agricultural household registration	1597	36.870	100.000	607	28.320	100.000
Marital status	Unmarried	39	0.900	0.900	67	3.130	3.130
	Married	4292	99.100	100.000	2076	96.870	100.000
Locality	East	1714	39.580	39.580	726	33.880	33.880
	Middle	1578	36.440	76.010	695	32.430	66.310
	West	1039	23.990	100.000	722	33.390	100.000
Schooling	No formal education	274	6.330	6.330	149	6.950	6.950
	Private school and literacy class	25	0.580	6.900	9	0.420	7.370
	Primary school	943	21.770	28.680	484	22.590	29.960
	Junior school	1574	35.720	64.400	757	35.320	65.280
	Vocational high school	71	1.640	66.040	23	1.070	66.360
	General high school	566	13.070	79.100	282	13.160	79.510
	Technical secondary school	223	5.150	84.250	92	4.290	83.810
	Technical school	39	0.900	85.150	16	0.750	84.550
	Associate degree (adult higher education)	120	2.770	87.920	87	4.060	88.610
	Associate degree (regular higher education)	213	4.920	92.840	87	4.060	92.670
	Bachelor’s degree (adult higher education)	84	1.940	94.780	59	2.750	95.430
	Bachelor degree (regular higher education)	195	4.500	99.280	90	4.200	99.630
	Graduate student or above	31	0.720	100.000	8	0.370	100.000
Social hierarchy	1 point	256	5.910	5.910	215	10.030	10.030
	2 points	295	6.810	12.720	148	6.910	16.940
	3 points	641	14.800	27.520	254	11.850	28.790
	4 points	769	17.760	45.280	296	13.810	42.600
	5 points	1540	35.560	80.840	771	35.980	78.580
	6 points	485	11.200	92.030	259	12.090	90.670
	7 points	205	4.730	96.770	97	4.530	95.190
	8 points	96	2.220	98.980	60	2.800	97.990
	9 points	14	0.320	99.310	12	0.560	98.550
	10 points	30	0.690	100.000	31	1.450	100.000

### Traditional gender concepts and physical exercise of different characteristic groups

Univariate analyses identified significant demographic disparities in tradiotional gender concepts and physical exercise between 2013 and 2023. For tradiotional gender concepts, a notable shift occurred across life stages: middle-aged adults (45–59 years old) held the most traditional views in 2013, while older adults (60 years old and above) became holding the most traditional gender concepts group in 2023. Non-agricultural household registration holders, eastern regions individuals, and those with higher education consistently reported more open attitudes across both years. For physical exercise, all subgroups showed substantial increases from 2013 to 2023, with non-agricultural household registration holders, married participants, higher educational attainment women and individuals with higher social hierarchy scores reporting significantly higher physical exercise rates in 2023 (Table 5).

**Table 5:** Traditional gender concepts and physical exercise among different demographic groups

Variable	Classification	2013		2023	
		Traditional gender concepts	Physical exercise (%)	Traditional gender concepts	Physical exercise (%)
Life stage	Young (15–44 years old)	42.455^***^	44.460^***^	29.218^***^	64.808^***^
	Middle-aged (45–59 years old)	47.340^***^	44.048^***^	42.714^***^	55.764^***^
	Older (60 years old and above)	21.175^***^	40.484^***^	50.187^***^	47.092^***^
Hukou	Agricultural household registration	51.637^***^	28.676^***^	45.828^***^	47.070^***^
	Non-agricultural household registration	42.816^***^	65.936^***^	34.071^***^	72.782^***^
Marital status	Unmarried	47.377	74.359^***^	44.988	58.209
	Married	48.394	42.125^***^	42.417	54.287
Locality	East	47.478^***^	56.359^***^	38.571^***^	62.121^***^
	Middle	49.340^***^	34.094^***^	45.723^***^	52.518^***^
	West	48.428^***^	32.050^***^	43.342^***^	48.476^***^
Schooling	No formal education	55.804^***^	17.153^***^	54.262^***^	23.649^***^
	Private school and literacy class	47.324^***^	48.000^***^	61.586^***^	22.222^***^
	Primary school	54.046^***^	22.375^***^	53.251^***^	40.289^***^
	Junior school	49.672^***^	37.492^***^	43.290^***^	52.576^***^
	Vocational high school	44.852^***^	56.338^***^	38.079^***^	69.565^***^
	General high school	46.056^***^	52.827^***^	36.554^***^	63.121^***^
	Technical secondary school	41.061^***^	65.022^***^	34.102^***^	59.783^***^
	Technical school	38.214^***^	69.231^***^	32.067^***^	75.000^***^
	Associate degree (adult higher education)	40.876^***^	81.667^***^	31.818^***^	77.011^***^
	Associate degree (regular higher education)	41.019^***^	63.380^***^	24.428^***^	81.609^***^
	Bachelor degree (adult higher education)	37.407^***^	82.143^***^	26.137^***^	86.441^***^
	Bachelor degree (regular higher education)	38.469^***^	75.385^***^	27.518^***^	85.556^***^
	Graduate student or above	35.034^***^	87.097^***^	17.851^***^	100.000^***^
Social hierarchy	1 point	50.362^***^	29.297^***^	50.999^***^	38.605^***^
	2 points	49.606^***^	30.508^***^	45.628^***^	47.297^***^
	3 points	50.764^***^	30.889^***^	44.892^***^	47.244^***^
	4 points	49.427^***^	37.971^***^	40.979^***^	51.014^***^
	5 points	48.159^***^	46.104^***^	41.346^***^	56.809^***^
	6 points	44.159^***^	54.021^***^	38.708^***^	66.795^***^
	7 points	45.307^***^	60.000^***^	34.544^***^	65.979^***^
	8 points	45.763^***^	63.542^***^	38.776^***^	70.000^***^
	9 points	40.280^***^	85.714^***^	50.565^***^	66.667^***^
	10 points	55.042^***^	46.667^***^	52.742^***^	54.839^***^

*shows significance at *p* < 0.05

**shows significance at *p* < 0.01

***shows significance at *p* < 0.001

**Fig. 1:**
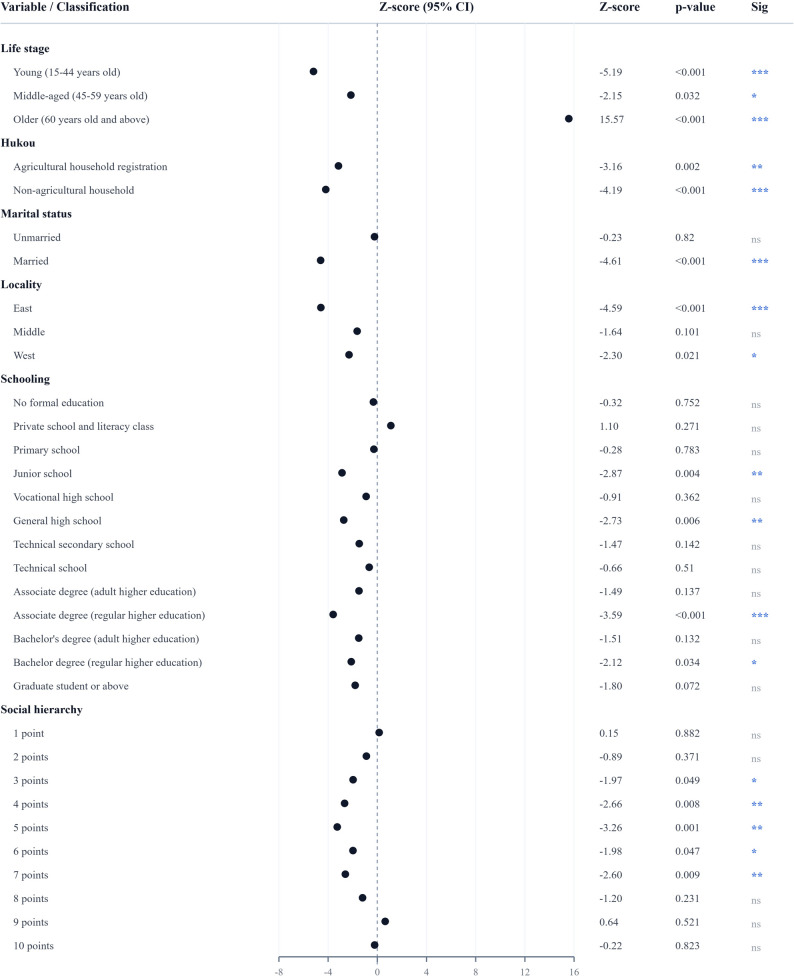
The Z-score forest plot of traditional gender concepts. Statistical analysis indicators: ns shows shows not significant; * significance at *p* < 0.05; ** shows significance at *p* < 0.01; *** shows significance at *p* < 0.001

The Z-score forest plot confirmed significant group differences in traditional gender concepts from 2013 to 2023 (Fig. 1). Young (Z = -5.19, *p* < 0.001) and middle-aged (Z = -2.15, *p* < 0.05) adults exhibited more open and modern gender concepts, whereas older adults (Z = 15.57, *p* < 0.001) held more traditional concepts. Both agricultural (Z = -3.16, *p* < 0.01) and non-agricultural (Z = -4.19, *p* < 0.001) household registration groups, married participants (Z = -4.61, *p* < 0.001), and those from eastern (Z = -4.59, *p* < 0.001) and western (Z = -2.30, *p* < 0.05) regions reported more open gender concepts from 2013 to 2023. Individuals with junior high school, general high school, associate degree (regular higher education), and bachelor degree (regular higher education), as well as middle-to-upper social hierarchy (3–7 points), demonstrated more open and modern gender concepts from 2013 to 2023.

**Fig. 2:**
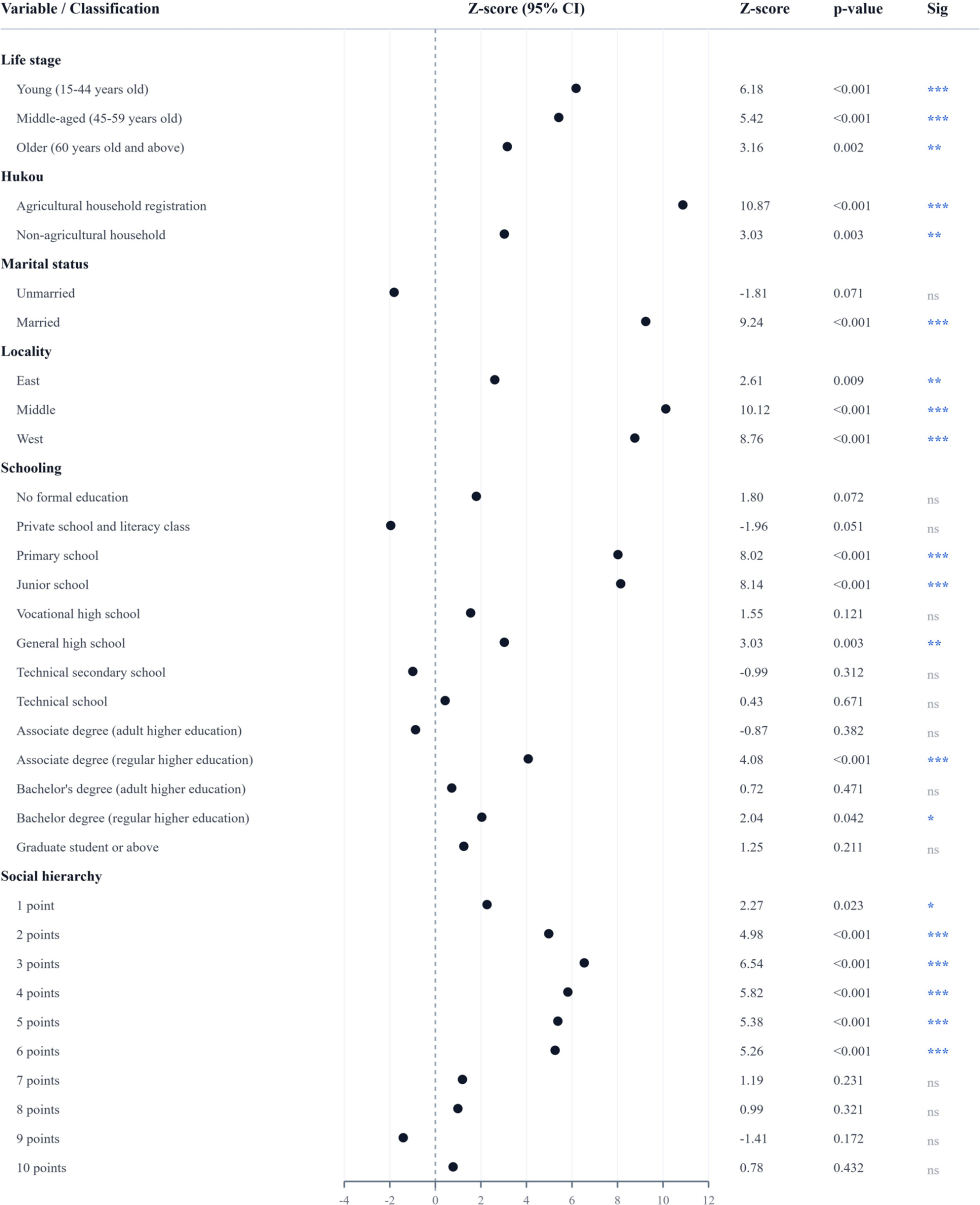
The Z-score forest plot of physical exercise. Statistical analysis indicators: ns shows shows not significant; * significance at *p* < 0.05; ** shows significance at *p* < 0.01; *** shows significance at *p* < 0.001

For physical exercise, the Z-score forest plot revealed widespread, significant increased from 2013 to 2023 (Fig. 2). All life stages showed higher participation (young: Z = 6.18, *p* < 0.001; middle-aged: Z = 5.42, *p* < 0.001; older: Z = 3.16, *p* < 0.01). Agricultural (Z = 10.87, *p* < 0.001) and non-agricultural (Z = 3.03, *p* < 0.01) household registration holders, married participants (Z = 9.24, *p* < 0.001), and all regions (east: Z = 2.61, *p* < 0.01; middle: Z = 10.12, *p* < 0.001; west: Z = 8.76, *p* < 0.001) exhibited higher physical exercise participation rate, with the midddle region showing the strongest effect. Participants with primary school, junior school, general high school, associate degree (regular higher education), and bachelor degree (regular higher education) (*p* < 0.05 to *p* < 0.001), as well as lower-to-middle social hierarchy (1–6 points), reported significantly increased physical exercise participation rate.

### The influence of physical exercise on traditional gender concepts

Table 6 presented the OLS regression results examining how physical exercise predicts traditonal gender concepts, with separate models for 2013 and 2023, and progressive inclusion of control variables. Across all models, physical exercise demonstrated a consistently negative and highly significant association with traditional gender concepts. In 2013, the effect size ranges from β = -7.216 (*p* < 0.001, Model 1) to β = -3.245 (*p* < 0.001, Model 2) after adjusting for covariates. In 2023, the negative association remained robust, with estimates from β = -10.957 (*p* < 0.001, Model 3) to β = -4.259 (*p* < 0.001, Model 4). This indicated that individuals who participated in physical exercise hold significantly more open and modern gender concepts, and this relationship strengthend in magnitude from 2013 to 2023.

**Table 6:** OLS estimation results of the impact of physical exercise on traditional gender concepts

Variables	2013		2023		
	Model 1	Model 2	Model 3	Model 4	Model 5
Participation (Not participation = 0)	-7.216^***^(0.614)	-3.245^***^(0.651)	-10.957^***^(1.046)	-4.259^***^(1.038)	-2.532^***^(0.668)
Life stage (Young (15–44 years old)					
Middle-aged (45–59 years old)		4.350^***^(0.891)		10.109^***^(1.303)	
Older (60 years old and above)		6.983^***^(0.907)		17.243^***^(1.342)	
Life stage					5.219^***^(0.360)
Non-Agricultural household registration (Agricultural household registration = 0)		-5.660^***^(0.724)		-7.284^***^(1.166)	
Hukou					-6.658^***^(0.629)
Married (Unmarried = 0)		-2.096(3.034)		-0.191(2.592)	
Marital status					-1.802(1.984)
Locality (East = 1)					
Middle		-2.006^**^(0.681)		3.530^**^(1.212)	
West		-2.782^***^(0.777)		-0.516(1.230)	
Locality					-0.815^**^(0.333)
Schooling (No formal education = 1)					
Private school and literacy class		-5.749(4.641)		4.834(6.823)	
Primary school		-0.380(1.209)		2.379(2.055)	
Junior school		-3.017^*^(1.216)		-3.204(2.020)	
Vocational high school		-6.007^*^(2.610)		-2.954(4.527)	
General high school		-5.296^***^(1.412)		-9.158^***^(2.220)	
Technical secondary school		-8.398^***^(1.785)		-8.964^**^(2.976)	
Technical school		-11.144^***^(3.052)		-11.788^**^(4.155)	
Associate degree (adult higher education)		-7.088^**^(2.301)		-9.859^***^(2.816)	
Associate degree (regular higher education)		-6.894^***^(1.902)		-13.496^***^(2.934)	
Bachelor degree (adult higher education)		-10.227^***^(2.822)		-11.010^***^(3.283)	
Bachelor degree (regular higher education)		-8.648^***^(1.991)		-9.627^***^(2.831)	
Graduate student or above		-11.601^**^(3.873)		-12.665(6.612)	
Schooling					-1.116^***^(0.111)
Social hierarchy (1 point = 1)					
2 points		-0.694(1.627)		-3.585^*^(2.469)	
3 points		0.498(1.441)		-4.647^*^(2.052)	
4 points		0.611(1.415)		-6.599^***^(1.991)	
5 points		0.515(1.357)		-5.317^**^(1.726)	
6 points		-2.057(1.606)		-5.421^**^(2.069)	
7 points		-0.386(1.882)		-8.041^**^(2.827)	
8 points		-0.850(2.401)		-5.842*(3.270)	
9 points		-5.828(6.147)		3.463(4.662)	
10 points		5.676(4.312)		1.039(5.252)	
Social hierarchy					-0.223(0.150)
Year2023					-3.184^***^(0.764)
Physical exercise x Year23					-3.954^***^(1.077)
_cons	51.445^***^(0.379)	54.068^***^(3.539)	48.459^***^(0.803)	44.274^***^(3.718)	49.725^***^(2.488)
*N*	4331	4331	2143	2143	6474
*R* ^2^	0.032	0.103	0.049	0.230	0.154
F	137.982	17.849	109.656	26.394	39.020

*shows significance at *p* < 0.05

**shows significance at *p* < 0.01

***shows significance at *p* < 0.001

Compared to the young (15–44 years old), older adults (60 years old and above) exhibited significantly more traditional gender concepts in both years (e.g., 2013: β = 6.983, *p* < 0.001; 2023: β = 17.243, *p* < 0.001), with a notably larger effect in 2023. Middle-aged adults (45–59 years old) also showed significantly more traditional gender concepts in 2023 (β = 10.109, *p* < 0.001). Non-agricultural household registration status was associated with more open and equal gender concepts in 2013 (β = -5.660, *p* < 0.001), and this effect remained consistent with more open and equal gender concepts in 2023 (β = -7.284, *p* < 0.001). In 2013, middle (β = -2.006, *p* < 0.01) and western (β = -2.782, *p* < 0.001) regions showed more open and modern gender concepts relative to the east; in 2023, only the middle region retains a significant effect (β = 3.530, *p* < 0.01), indicating more traditional concepts. In 2013, the majority of educational attainment categories (such as technical school, associate degrees, and bachelor’s degrees) exhibited statistically significant negative coefficients, indicating that individuals with higher levels of education demonstrated more progressive gender concepts. This pattern persisted in 2023, with stronger negative effects observed for regular higher education degrees (e.g., associate degree: β = -9.859, *p* < 0.001; bachelor’s degree: β = -11.010, *p* < 0.001). The variable was measured on a 1–10 self-ranking scale, where 1 represents the lowest subjective social position and 10 the highest. Scores 2–8 correspond to respondents who perceived themselves as belonging to lower-middle to middle social hierarchy. Interestingly, particulaly in 2023, the social hierarchy mainly among women with scores of 2–8 demonstrated a significant equality in gender concepts, which indicates that individuals within this range scored significantly higher on gender egalitarian concepts compared with those who held significantly more egalitarian gender concepts than those at the lowest (score = 1) and highest (scores 8–10) ends of the scale.

To account for potential secular trends and period effects (e.g., societal modernization) and examine whether the association between physical exercise and traditional gender concepts strengthened over time, we pooled the 2013 and 2023 datasets and estimated an interaction model. The coefficient for Year2023 was significantly negative, confirming substantial temporal changes in traditional gender concepts consistent with broader societal modernization. After controlling for these overall temporal shifts, the interaction term between physical exercise and Year2023 was significantly negative. This result indicates that the strengthening association between physical exercise and traditional gender concepts from 2013 to 2023 is not driven by general secular trends, but reflects a genuine increase in the focal relationship net of period effects.

### Propensity score matching estimates of physical exercise on gender concepts

Classical OLS regression yielded inconclusive findings regarding the relationship between physical exercise and traditional gender concepts among women, primarily due to the presence of substantial selection bias in physical exercise. To address this endogeneity issue and isolate the effect of physical exercise by controlling for confounding variables, we employed three core PSM approaches: nearest neighbor matching, radius matching, and kernel matching. Prior to effect estimation, a balance test was conducted on all covariates post-matching. All variables achieved satisfactory balance, confirming that the PSM method effectively eliminated systematic differences in observable characteristics between women who participated in physical exercise and those who did not, thus validating the reliability of subsequent estimates. The PSM results revealed high consistency across the three matching strategies, with all estimates significant at the 1% statistical level. Aggregating the average treatment effects on the treated across the three methods, we found that physical exercise exerted a significant negative effect on traditional gender concepts score in both years. Specifically, participation in physical exercise was associated with a decrease of 4.200 points in traditional gender concepts score in 2013, and a reduction of 2.850 points in 2023. These results, derived after adjusting for selection bias, offer strong evidence that physical exercise positively influences the development of open and egalitarian gender concepts.

### Endogenous treatment

To address potential endogeneity in the relationship between physical exercise and traditional gender concepts, we emploied IV-2SLS approach. The instruments employed in this study are as follows: (1) IV-2SLS: the instrumental variable for the IV-2SLS approach is “watching sports games on the spot”; (2) IV-2SLS: the instrumental variable for the IV-2SLS method is “going to gymnasium or gym for exercise”; and (3) IV-2SLS: the IV-2SLS model incorporates both instrumental variables simultaneously. Both instrumental variables capture sports-related behaviors that are conceptually and empirically correlated with physical exercise. Individuals who watch sports games on the spot are likely to be sports enthusiasts with higher propensity to participate in physical exercise. Similarly, those who go to gymnasium or gym for exercise directly reflect fitness-oriented lifestyle choices. First-stage regression results confirm that both instruments variables are highly significant predictors of physical exercise (*p* < 0.01). We argue that both instruments variables affect traditional gender concepts only through physical exercise, not through direct channels. While both instruments are sports-related, they satisfy the exclusion restriction for the following reasons: (a) “watching sports games on the spot” is a spectator behavior that reflects interest in sports entertainment rather than direct participation in gender socialization; (b) “going to gymnasium or gym for exercise” is a facility access variable that captures availability and preference for structured fitness environments; (c) conditional on physical exercise and other controls, residual variation in both instruments reflects leisure consumption patterns and local sports infrastructure rather than systematic differences in gender norms. Instrument validity was confirmed by the weak instrument test, with F-statistics ranging from 280.514 to 415.649 (all *p* < 0.001), far exceeding the critical value of 10, rejecting the null hypothesis of weak instruments (Table 7). Across all three specifications in 2013, the coefficient of physical exercise was negative but statistically insignificant (ranging from − 0.564 to -0.664, with standard errors between 2.235 and 2.927; *p* > 0.05). The Durbin–Wu–Hausman test yielded a p-value of 0.876, indicating no significant difference between OLS and IV-2SLS estimates. This suggested that the significant negative correlation observed in OLS was driven by endogeneity biases rather than a genuine inhibition effect of physical exercise on traditional gender concepts in 2013. For the 2023 sample, the weak instrument test also confirmed instrument validity (F-statistics: 125.868 to 239.005, *p* < 0.001). In sharp contrast to 2013, the coefficient of physical exercise was consistently negative and highly significant across all specifications, ranging from − 12.060 (*p* < 0.001) to -16.790 (*p* < 0.001) (Table 8). The Durbin–Wu–Hausman test was significant (*p* < 0.05), indicating that OLS estimates were biased downward due to unobserved confounding factors. These results suggested that after correcting for endogeneity, physical exercise was associated with variations in traditional gender concepts in 2023.

**Table 7:** Results of the IV-2SLS (2013)

Categories	(1)	(2)	(3)
	IV-2SLS	IV-2SLS	IV-2SLS
Participation (Not participation = 0)	-0.664(2.843)	-0.564(2.927)	-0.615(2.235)
Life stage (Young (15–44 years old = 1))			
Middle-aged (45–59 years old)	4.303^***^(0.892)	4.301^***^(0.894)	4.302^***^(0.892)
Elderly (60 years old and above)	6.922^***^(0.911)	6.920^***^(0.911)	6.921^***^(0.910)
Non-Agricultural household registration (Agricultural household registration = 0)	-6.221^***^(0.942)	-6.243^***^(0.956)	-6.232^***^(0.862)
Married (Unmarried = 0)	-1.420(3.122)	-1.394(3.135)	-1.407(3.092)
Locality (East = 1)			
Middle	-1.760^**^(0.728)	-1.750^**^(0.733)	-1.755^**^(0.709)
West	-2.506^***^(0.830)	-2.495^***^(0.823)	-2.501^***^(0.803)
Schooling (No formal education = 1)			
Private school and literacy class	-6.235(4.720)	-6.254(4.726)	-6.244(4.711)
Primary school	-0.454(1.211)	-0.457(1.211)	-0.455(1.210)
Junior school	-3.370^***^(1.279)	-3.383^***^(1.272)	-3.376^***^(1.251)
Vocational high school	-6.652^**^(2.688)	-6.677^**^(2.691)	-6.664^**^(2.651)
General high school	-5.874^***^(1.538)	-5.897^***^(1.540)	-5.885^***^(1.484)
Technical secondary school	-9.171^***^(1.989)	-9.201^***^(1.983)	-9.186^***^(1.909)
Technical school	-11.88^***^(3.154)	-11.91^***^(3.130)	-11.89^***^(3.099)
Associate degree (adult higher education)	-8.267^***^(2.583)	-8.313^***^(2.674)	-8.289^***^(2.493)
Associate degree (regular higher education)	-7.582^***^(2.039)	-7.608^***^(2.057)	-7.595^***^(1.989)
Bachelor degree (adult higher education)	-11.340^***^(3.106)	-11.390^***^(3.082)	-11.36^***^(2.992)
Bachelor degree (regular higher education)	-9.499^***^(2.221)	-9.531^***^(2.196)	-9.515^***^(2.126)
Graduate student or above	-12.66^***^(4.024)	-12.70^***^(4.010)	-12.68^***^(3.948)
Social hierarchy (1 point = 1)			
2 points	-0.767(1.627)	-0.770(1.629)	-0.769(1.627)
3 points	0.425(1.444)	0.422(1.446)	0.423(1.444)
4 points	0.489(1.422)	0.484(1.424)	0.487(1.420)
5 points	0.279(1.378)	0.269(1.386)	0.274(1.372)
6 points	-2.334(1.630)	-2.345(1.646)	-2.340(1.626)
7 points	-0.807(1.924)	-0.823(1.924)	-0.815(1.901)
8 points	-1.439(2.499)	-1.462(2.493)	-1.451(2.461)
9 points	-7.060(6.276)	-7.107(6.269)	-7.083(6.212)
10 points	5.438(4.309)	5.429(4.328)	5.433(4.315)
_cons	53.000^***^(3.713)	52.96^***^(3.731)	52.98^***^(3.645)
First-stage F statistic	415.649^***^	280.514^***^	299.252^***^
Durbin–Wu–Hausman Test	0.876	0.894	1.539
N	4331	4331	4331
r2_a	0.094	0.094	0.094

* shows significance at *p* < 0.05

** shows significance at *p* < 0.01

*** shows significance at *p* < 0.001

**Table 8:** Results of the IV-2SLS (2023)

Categories	(1)	(2)	(3)
	IV-2SLS	IV-2SLS	IV-2SLS
Engagement (Not engagement = 0)	-12.060^***^(3.907)	-16.790^***^(5.000)	-13.75^***^(3.419)
Life stage (Young (15–44 years old = 1))			
Middle-aged (45–59 years old)	9.932^***^(1.326)	9.824^***^(1.360)	9.893^***^(1.335)
Elderly (60 years old and above)	16.560^***^(1.417)	16.150^***^(1.467)	16.41^***^(1.414)
Non-Agricultural household registration (Agricultural household registration = 0)	-6.023^***^(1.315)	-5.257^***^(1.443)	-5.749^***^(1.290)
Married (Unmarried = 0)	-0.775(2.617)	-1.131(2.676)	-0.903(2.628)
Locality (East = 1)			
Middle	3.494^***^(1.22)	3.473^***^(1.245)	3.486^***^(1.228)
West	-0.687(1.235)	-0.792(1.255)	-0.725(1.239)
Schooling (No formal education = 1)			
Private school and literacy class	4.854(6.718)	4.866(6.755)	4.859(6.725)
Primary school	3.533^*^(2.119)	4.234^*^(2.216)	3.784^*^(2.117)
Junior school	-1.435(2.168)	-0.360(2.334)	-1.050(2.146)
Vocational high school	0.070(4.723)	1.907(4.961)	0.727(4.694)
General high school	-6.779^***^(2.507)	-5.334^*^(2.745)	-6.262^**^(2.464)
Technical secondary school	-6.945^**^(3.170)	-5.719^*^(3.368)	-6.507^**^(3.168)
Technical school	-8.564^*^(4.452)	-6.604(4.861)	-7.863^*^(4.460)
Associate degree (adult higher education)	-6.702^**^(3.222)	-4.784(3.515)	-6.016^*^(3.151)
Associate degree (regular higher education)	-10.130^***^(3.324)	-8.088^**^(3.710)	-9.401^***^(3.274)
Bachelor degree (adult higher education)	-7.338^**^(3.702)	-5.106(4.181)	-6.540^*^(3.674)
Bachelor degree (regular higher education)	-6.227^*^(3.212)	-4.161(3.563)	-5.488^*^(3.136)
Graduate student or above	-8.754(6.746)	-6.377(6.969)	-7.904(6.696)
Social hierarchy (1 point = 1)			
2 points	-2.979(2.505)	-2.612(2.556)	-2.848(2.513)
3 points	-4.076^*^(2.082)	-3.729^*^(2.116)	-3.952^*^(2.083)
4 points	-5.972^***^(2.013)	-5.591^***^(2.051)	-5.836^***^(2.013)
5 points	-4.372^**^(1.790)	-3.797^**^(1.833)	-4.166^**^(1.774)
6 points	-4.092^*^(2.178)	-3.285(2.220)	-3.803^*^(2.144)
7 points	-6.794^**^(2.925)	-6.037^**^(3.013)	-6.524^**^(2.923)
8 points	-4.045(3.483)	-2.953(3.624)	-3.654(3.475)
9 points	5.124(4.316)	6.133(4.164)	5.485(4.213)
10 points	2.315(5.379)	3.090(5.483)	2.592(5.396)
_cons	46.440^***^(3.892)	47.760^***^(4.046)	46.91^***^(3.872)
First-stage F statistic	239.005***	125.868***	159.347***
Durbin–Wu–Hausman Test	4.359*	7.077**	8.693**
N	2143	2143	2143
r2_a	0.198	0.163	0.187

* shows significance at *p* < 0.05

** shows significance at *p* < 0.01

*** shows significance at *p* < 0.001

## Discussion

### Summary of key findings

The subjects of the investigation mainly focused on middle-aged and older women, those with rural household registration, married women, those with primary, junior school or general high school education bakground, and women from the middle social hierarchy. In order to explore the frequency of women participation in physical exercise over the past decade, the statistics showed that in 2013, the number of women participation in physical exercise was less than those who did not, while in 2023, the participation rate of women in physical exercise significantly increased and was higher than that of those who did not participate. Specifically, from 2013 to 2023, women of all ages who were married and registered in either agricultural or non-agricultural areas, came from the middle, eastern, and western regions, possess educational backgrounds ranging from primary school, junior school, general high school, associate degree (regular higher education), up to bachelor’s degree (regular higher education), and belonged to social hierarchy levels 1 through 6, demonstrated a notable increase in their participation in physical exercise. Furthermore, there was a significant reduction in traditional gender concepts among young and middle-aged women, regardless of whether they were registered in rural or urban areas. This decline was also seen among married women, women from both eastern and western regions, those with educational levels from junior school, general high school, associate degree (regular higher education), to bachelor’s degree (regular higher education), as well as women in social hierarchy levels 3 to 7 between 2013 and 2023. To investigate the connection between physical exercise and traditional gender concepts, the statistical findings showed that physical exercise was able to reduce the scores of traditional gender concepts in both 2013 and 2023, with a greater decrease observed in 2023 compared to 2013. In comparison with agricultural household registration, non-agricultural household registration exerted a considerable negative influence on traditional gender concepts. And the middle-aged and (45–59 years old) older (60 years old and above) women had positive relationship with traditional gender concepts, which indicated that middle-aged (45–59 years old) and older (60 years old and above) women in the middle region hold pronounced in traditional gender concepts. When compared with the eastern egions, the middle region has experienced relatively slower economic transformation and remains more reliant on agriculture and traditional manufacturing [46]. Such economic structures tend to preserve conventional social norms and family values, which may strengthen traditional attitudes. Also, the middle region is characterized by large-scale labor outmigration to developed regions. The relatively high proportion of elderly and left-behind populations, who are more inclined to adhere to traditional norms, together with the outflow of younger and more liberal individuals, contributes to the persistence of traditional views [47, 48]. In addition, the middle region has a long-standing and deeply rooted cultural heritage that emphasizes traditional family ethics and social conventions. These cultural legacies are transmitted across generations and maintain strong continuity independent of short-term economic fluctuations [49]. While non-agricultural household registration, high education backgroud, and midddle social hierarchy were negatively correlated with traditional gender concepts. The recent increase in women education and the subsequent change in attitudes towards assortative marriages may alter this trend [50]. Higher educational attainment among women brought advantages in the workplace. A positive and open gender concepts are supported by female gender, younger age, urban living, higher income, and higher levels of maternal and paternal education [51]. Besides, higher education background have been shown to be associated with greater cultural capital, which has been demonstrated to enable the transformation of the experience of equality on the sports field into ideologies and practices of equality in social life. Those who are young and lived in a city, have a full-time job and reported a better physical condition, as well as having enough income to meet their daily needs, are more likely to participation in physical exercise regularly.

### Interpretation of the central causal relationship and its evolution from 2013 to 2023

From a theoretical standpoint, sport has traditionally been an important arena for shaping and expressing gender identities [18], and it carries positive potential for empowering women and reshaping gender norms [19]. Physical exercise can serve to develop a critical awareness of gender regulation [33, 34]. Participating in physical exercise encourages women to go beyond their physical and mental boundaries. Their achievements in surpassing conventional gender norms also empower them to confront existing stereotypes regarding women’s physical and mental abilities. This process clearly illustrates how participation in physical exercise can effectively reshape and subvert traditional gender concepts in 2023 compared to 2013 in this study. Despite numerous positive advancements in gender equality over recent decades, women remain underrepresented in positions of power and prestige, while continuing to bear a disproportionate share of unpaid domestic labor [52]. Even when men and women exhibit similar characteristics, preferences, and ambitions, the differing views and stereotypical expectations held by others consign them to distinct spheres [53]. It is reflected at a structural level in the under-representation of women in senior positions and at a cultural level in the legitimacy of a wide range of practices to value men and to facilitate their access to such positions and to undervalue women and to inhibit their access [54]. However, from the Confucianism of the Imperial era to contemperary China, traditional gender concepts begin at an extreme during the past century [21]. Historical developments have contested traditional notions of gender, leading to the widespread acceptance of contemporary principles of gender equality. Women are no longer subordinate to men. Modern women can hold their own and play a significant role in social production and labor, even accomplishing tasks that men cannot. The study further underscores that economic development and gender equality in rights proceed in tandem with the reconfiguration, rather than the repression, of gender norms, giving rise to new and more egalitarian forms of cross-gender social differentiation [55]. Physical exercise empowers women to transcend physical and mental constraints, which in turn helps them transcend traditional gender concepts and challenge dominant stereotypes regarding women’s capabilities in 2023 compared with 2013. The impact of physical exercise on traditional gender concepts has been significantly shaped by different environmental and policy elements from 2013 to 2023. The increase in the effect size from 2013 to 2023 should be interpreted as a period-related change, potentially shaped by societal modernization and gender culture trends, rather than purely a strengthening of the exercise–gender concept link. By including a Year dummy and Exercise×Year interaction in the pooled model, we explicitly separated general temporal trends from changes in the focal association. Results indicate that the temporal change in the relationship remains observable after accounting for secular societal shifts, supporting the robustness of our main conclusion while acknowledging the role of period effects.

### Contextualizing the rise in women’s exercise

The significant physical exercsie increase in the participation rate of Chinese women is the result of the combined effect of five factors: policy promotion, resource expansion, awakening of women’s consciousness, optimization of market supply, and innovation of social concepts. The National Fitness Program and the Outline for the Development of Chinese Women have incorporated women’s physical exercise into the national strategy, clearly stating that “all-round development of women’s sports should be promoted”. Physical exercise fiscal expenditure has continued to increase since 2013, Healthy China 2030 lists women as a key group, promotes the popularization of the concept that “exercise promotes health”, and lowers the institutional threshold for women to participate in sports from the top-level design. The sports facilities have become more widespread. Community fitness paths, park walkways, and women-only fitness areas have been built in large numbers, solving the problem of “where to exercise”. The sports events and scenarios have become more diverse, the number of women sports communities has grown rapidly, and the participation barriers have been lowered. Under the dual pressures of work and family, exercise has become an important way for women to manage their health, relieve anxiety and improve their posture, health anxiety and the “active health” awareness have replaced passive health care. The self-worth and identity of women have been upgraded. The sports have shifted from “weight loss and body shaping” to pursuing a sense of strength, control and achievement, becoming a way for women to break through gender stereotypes and express themselves. In addition, the market supply precisely caters to the needs of women, and fitness institutions offer courses that are friendly to women (such as pregnancy and postpartum recovery, as well as women’s strength training), addressing both physiological and experiential pain points. Meanwhile, gender stereotypes have been weakened, concepts such as “women are not suitable for intense exercise” and “exercise is in opposition to elegance” have been challenged, women strength and healthy beauty have become the mainstream aesthetic, men are taking on more household chores and the idea of sharing parenting responsibilities has become more widespread, this has reduced the time women spend on sports and the resistance from their families and society’s tolerance and recognition for women’s sports have significantly increased.

### Theoretical implications and mechanism exploration

Embodiment Theory posits that cognition and ideology are shaped by bodily experiences thus, intentional, repetitive physical exercise serves as a critical vehicle to reshape gender-related cognition, challenge entrenched gender norms, and foster egalitarianism. Grounding the analysis in this framework clarifies how physical activity drives ideological change, addressing the identified gap. For women, physical exercise directly confronts the deep-seated stereotype equating femininity with weakness and passivity—a norm restricting self-perception and social participation. Active bodily participation helps women disentangle their self-image from gendered bodily expectations. Consistent exercise reinforces bodily empowerment: enhancing perceived bodily control, reducing adherence to restrictive gender norms, and prompting critical questioning of broader traditional gender concepts. This aligns with embodiment theory, which frames bodily practices as agents of cognitive and ideological transformation. Throughout the exercise process, individuals progressively increase their self-efficacy through the consistent attainment of exercise objectives, such as maintaining training persistence and surpassing physical limits. This enhancement of self-efficacy subsequently reinforces the individual’s sense of autonomy, challenges the conventional notion of “passive compliance,” and consequently diminishes the impact of traditional beliefs. The integration of these two elements offers an explanatory framework for the transformation of sports into ideology through the lens of the “interaction between body and mind,” thereby facilitating a more comprehensive understanding of the phenomenon. Existing studies have explored the correlation between physical exercise and gender attitude changes [18, 19], women are more focused on their own physical condition and use physical exercise to break free from the traditional gender constraints. To verify the relationship between physical exercise and traditional gender concepts, an IV-2SLS approach was employed. This method demonstrated that physcal exercise exerts a significant inhibition impact on such traditional gender concepts. When it refers to “watching sports games on the spot”, which can enhance individual willingness to participate in sports through “atmosphere infection (such as interactive experiences of group viewing)” and “lowering the participation threshold (such as being inspired by the event to try related sports)”. Furthermore, “going to gymnasium or gym for exercise” promotes sports participation behavior directly through “hardware support (professional facilities to reduce sports costs)” and “social networks (interaction among like-minded people in the gym)”. Existing reviews and empirical studies consistently show that attendance at on-site sports events (atmosphere influence, participation motivation) and the use of professional venues/gyms (facility accessibility, environmental drive) are strong exogenous/quasi-exogenous factors of physical exercise [56, 57]. Theoretically, the core determinants of gender cocnepts are family upbringing, educational level and social and cultural norms [58], venue/event contact is a specific behavioral choice, which does not carry gender concepts signal and does not directly affect gender concepts, satisfying the exclusivity constraint of instrumental variables. However, “watching sports events on the spot” and “going to the gym” are specific consumption/behavioral choices and do not inherently carry ideological orientation, individuals may choose to go to the gym for health and social purposes rather than to embrace egalitarianism. Watching the event on site might be out of interest and has no direct logical connection with gender ideology. The validity of the instrumental variables “watching sports games on the spot” and “going to gymnasium” relies on two key assumptions: relevance and exclusion. Regarding relevance, these variables are strongly predictive of individual physical exercise participation, as they reflect concrete opportunities and behaviors that directly encourage engagement in sports activities. For the exclusion restriction, we argue that these instruments represent specific, context-bound behavioral choices that do not directly signal preexisting gender concepts or gender-role attitudes. Unlike attitudinal variables, in-person sports attendance and gym visits are activity-focused behaviors that capture exposure to physical exercise environments rather than direct expressions of gender ideology. Potential confounding pathways—such as media exposure to athletic role models or social network effects—are limited in scope. Watching sports games on the spot involves in-person attendance rather than passive media consumption, reducing exposure to mediated gendered narratives. Similarly, while social interactions may occur in these settings, the theoretical mechanism linking the instruments to gender concepts operates primarily through the experience of physical exercise participation itself. Existing research supports the use of such context-specific activity measures as plausibly exogenous instruments for physical activity, as they are less likely to exert independent effects on gender attitudes outside of their influence on exercise behavior.

### Policy implications

The divergent findings between 2013 and 2023 highlight the evolving role of physical exercise in shaping traditional gender concepts. In 2013, gender barriers in physical exercise remained salient, and gender equality discourse was less pervasive, limiting the ability of physical exercise to reshape gender attitudes. By 2023, broader societal shifts—including expanded women physical exercise, increased gender equality education, and more inclusive athletic environments—had transformed sports into a robust vehicle for advancing egalitarian gender concepts, as reflected in the significant robustness check to alleviate potential endogeneity bias effect identified by IV-2SLS. The period 2013–2023 witnessed systematic governmental efforts to promote female sports participation and gender equality. The National Fitness Plan (2016–2020) explicitly designated women as a priority group, while the Healthy China 2030 Planning Outline (2016) mandated targeted physical health intervention programs for women alongside other specific demographic groups. The Sports Power Construction Outline (2019) further reinforced this commitment by requiring dedicated fitness plans for women. Most notably, the Outline for the Development of Chinese Women (2021–2030) issued by the State Council in September 2021 institutionalized gender-responsive sports policies, directing authorities to “guide and encourage women to participate in physical exercise” and “strengthen scientific guidance for women’s fitness activities”. This transformation is substantiated by concrete policy developments in China. These policy interventions collectively dismantled structural barriers that previously constrained women’s sports participation, thereby enabling physical exercise to function as an effective mechanism for gender attitude transformation by 2023 rather than merely reinforcing traditional divisions as observed in 2013.

### Limitations and future research

This study has several limitations. Firstly, physical exercise is operationalized as a binary indicator, which does not found for distinctions in exercise frequency, intensity, or type in CGSS database. As different forms of physical activity may be embedded in distinct social environments and reflect different gender norms, the effects of physical exercise may not be uniform across various types and intensities of exercise. For instance, occasional light exercise and regular high-intensity exercise may differ significant in the mechanism of influence and the magnitude of effect on traditional gender concepts. By grouping these heterogeneous groups into a single “participation groups” through binary measurement, we blurs the true effects of different types of exercise. This is not leading to averaging bias in the research results, making it impossible to accurately reflect the true association between physical exercise and the traditional gender concepts, but also hinders the effective control of confounding variables due to the failure to distinguish the specific dimensions of exercise. To address this limitation, future research will adopt more granular and comprehensive measurement strategies to capture the multi-dimensional characteristics of physical exercise. Specifically, two main approaches will be implemented: First, multi-dimensional measurement tools will be used to assess physical exercise in detail, including quantifying exercise frequency (e.g., times per week), duration (e.g., minutes per session), intensity (e.g., light, moderate, high intensity based on heart rate or self-report), and type (e.g., individual sports such as running, swimming vs. team sports such as basketball, volleyball). This will help to avoid the homogenization of heterogeneous exercise groups and accurately identify the differential effects of various exercise dimensions on gender attitudes. Second, group regression analysis will be employed to further subdivide the exercise participation group into different subgroups based on exercise type, intensity, or frequency. By separating the effects of different forms of exercise through subgroup analysis, future studies can more precisely test the relationship between physical exercise and gender attitudes, thereby making up for the measurement limitations of this study and providing more targeted theoretical and practical implications. In addition, This study only uses data from 2013 to 2023, and is unable to capture the continuous changing trends from 2013 to 2023. It may miss the impact of short-term policy shocks or social events. Moreover, key variables such as family gender division of labor, accessibility of community sports facilities, and media exposure were not included. These factors may simultaneously affect physical exercise and traditional gender concepts, resulting in a variable omission bias in the model. Even if individual and family characteristics are controlled, the interference of such confounding factors still cannot be completely excluded. These limitations should be addressed in future research through the implementation of follow-up surveys or the execution of dedicated specialised studies. Finally, and most importantly, in the mixed cross-sectional data, samples from different years are independently sampled, making it impossible to track the loss of the same population. At the same time, some key variables (such as social hierarchy and educational level) have missing values. Although list deletion processing is adopted, it may lead to sample selection bias and affect the robustness of the results. Building upon the 2023 findings, several future developments appear poised to further amplify the gender-transformative potential of physical exercise in China.

## Conclusion

This study investigated the effect of physical exercise on women’s traditional gender concepts in China using 2013 and 2023 survey data, applying IV-2SLS to address endogeneity. OLS results showed that physical exercise significantly reduces traditional gender concepts in both years, with a stronger negative impact in 2023. IV-2SLS estimations confirmed the robustness of this trend: while the 2013 effect was insignificant, the 2023 effect becomed significantly negative, indicating that physical exercise has weaken traditional gender concepts after accounting for endogeneity. Control variables including life stage, locality, and schooling consistently predict traditional gender concepts. These findings highlight the evolving role of physical exercise in promoting gender modernization in China, supporting policies to expand women’s physical exercise for gender equality.

## Data Availability

Analyses in the present study were conducted using publicly available datasets from the CGSS, with the official data repository available at the following URL:http://cgss.ruc.edu.cn/.
